# Amino Acid Mutations in Hemagglutinin-Neuraminidase Enhance the Virulence and Pathogenicity of the Genotype III Newcastle Disease Vaccine Strain After Intravenous Inoculation

**DOI:** 10.3389/fvets.2022.890657

**Published:** 2022-05-27

**Authors:** Xiaolong Lu, Xiaowen Liu, Qingqing Song, Xiaoquan Wang, Shunlin Hu, Xiufan Liu

**Affiliations:** ^1^Animal Infectious Disease Laboratory, College of Veterinary Medicine, Yangzhou University, Yangzhou, China; ^2^Jiangsu Co-innovation Center for Prevention and Control of Important Animal Infectious Diseases and Zoonosis, Yangzhou University, Yangzhou, China; ^3^Jiangsu Key Laboratory of Zoonosis, Yangzhou University, Yangzhou, China

**Keywords:** Newcastle disease virus, vaccine strain, virulence, HN, intravenous inoculation, reverse genetics technology

## Abstract

Newcastle disease virus (NDV), the causative agent that generally causes severe disease in poultry, continues to mutate and has thus evolved into 21 genotypes. We previously isolated a velogenic genotype III NDV JS/7/05/Ch that evolved from the vaccine strain Mukteswar, accompanying by amino acid mutations in Hemagglutinin-Neuraminidase (HN). Here, we sought to investigate the role of the mutant HN protein in NDV virulence. The HN genes of Mukteswar and JS/7/05/Ch were replaced reciprocally via reverse genetics, yielding two recombinant viruses rJS/MHN and rMu/JHN, respectively. rMu/JHN, in which the endogenous HN protein was replaced with the HN protein of JS/7/05/Ch, had a higher intravenous pathogenicity index (IVPI) value in chickens. Moreover, dual aa mutations (A494D and E495K from JS/7/05/Ch-type HN) were introduced into the HN protein of Mukteswar to generate the recombinant virus rMukHN494+495^JS^. This virus showed an equivalent IVPI value to that of rJS/7/05/Ch (generated from parental JS/7/05/Ch via reverse genetics). *In vitro* and *in vivo* assays further showed that A494D and E495K in HN induced antigenic changes, a higher replication level and a more intense inflammatory response. Taken together, these findings indicate that aa mutations in HN are crucial for the virulence of the genotype III Newcastle disease (ND) vaccine strain after intravenous inoculation. Our study further highlights that close surveillance is needed to monitor the genetic variation of ND vaccine strains.

## Introduction

Virulent Newcastle disease virus (NDV) strains generally cause the highly infectious and devastating disease in poultry (1). NDV, termed as avian paramyxovirus type 1 (APMV-1), is assigned as a member of the genus *Orthoavulavirus*, family *Paramyxoviridae* (2). NDV also belongs to the enveloped virus and contains a non-segmented, single-stranded, and negative-sense Ribonucleic Acid (RNA) (3). The substantial antigenic and genetic diversity of NDV has been widely recognized, although NDV has only one serotype. The latest classification criteria reveals that NDV consists of two subdivisions (class I and class II) classified into more than 20 genotypes. NDV can be divided into the avirulent, low (lentogenic), intermediate (mesogenic), and highly (velogenic) virulent strain via a range of virulence tests (4). Prior works have confirmed that the amino acid (aa) sequence at the fusion protein (F) cleavage site is a determining factor for NDV virulence. Generally, the cleavage site of mesogenic and velogenic NDVs is ^112^R/K-R-Q-R/K-R↓F^117^, while that of lentogenic NDVs is ^112^G/E-K/R-Q-G/E-R↓L^117^ (5). However, factors other than the F protein can also affect NDV virulence. Previous researches have explored the crucial effects of the hemagglutinin-neuraminidase protein (HN) on virulence and have demonstrated that NDVs carrying the identical F protein cleavage site can have different virulence (6–8). HN, a type II membrane glycoprotein, involves in regulating virulence, replication, and tissue tropism of NDV (9–11). Besides, the HN protein takes a main position in antigen recognition that carries seven important antigenic sites including site 1 to site 4, site 12, site 14, and site 23 (12).

ND has been an intractable problem since the first isolation of NDV in China in 1946 (13). Vaccination is the primarily method for controlling ND (14); live vaccines are widely used because of their strong immunogenicity. Common live ND vaccines used in China include La Sota, VGGA, and Mukteswar. The former two vaccines are lentogenic genotype II NDVs, while Mukteswar is a mesogenic genotype III NDV that is suitable for administration after 2 months of age. Vaccination has been extremely successful in controlling ND outbreaks; however, immune pressure imposed by frequent vaccination leads to emergence of viral variants. Although some countries have banned administration of mesogenic ND vaccines because of their potential pathogenicity, the attenuated Mukteswar clone is still used in China for its strong immunogenicity. In 2005, a virulent genotype III NDV (JS/7/05/Ch) with a high intravenous pathogenicity index (IVPI) was isolated, which shared more than 99% genomic identity with Mukteswar (15). This finding suggested that Mukteswar had evolved into a virulent NDV during vaccination.

To elucidate the molecular mechanisms responsible for the increased virulence of the genotype III NDV vaccine strain, we analyzed genomic differences between Mukteswar and JS/7/05/Ch and identified the critical protein and aa associated with NDV virulence.

## Materials and Methods

### Animals, Viruses, Cells, and Plasmids

Specific-pathogen-free (SPF) chickens and embryonated eggs were supplied by Merial Vital Laboratory Animal Technology Co., Ltd. (Beijing, China) and Zhejiang Lihua Agricultural Technology Co., Ltd (Zhejiang, China), respectively. The genotype III velogenic NDV JS/7/05/Ch isolated from a diseased chicken flock and the vaccine strain Mukteswar were kept by our lab. The viruses were amplified in allantoic fluid, collected under sterile conditions, and then stored at −70°C. The BSR-T7/5 cell line was provided by Dr. Zhigao Bu (Harbin Veterinary Institute, China). Peripheral blood mononuclear cells (PBMCs) were isolated from the whole blood of healthy SPF chickens using chicken PBMC separation medium (TBDscience, Tianjing, China). Three helper plasmids expressing the nucleocapsid (NP), phosphoprotein (P), and large polymerase (L) genes of NDV (pCI-NP, pCI-P, and pCI-L) were kept by our lab. The transcription vector TVT7R (0.0) was gifted from Dr. Andrew Ball (Alabama University, USA). pCR2.1 vector was purchased from Invitrogen (Carlsbad, CA, USA).

### RNA Extraction, Reverse Transcription-Polymerase Chain Reaction (RT-PCR), and Sequencing

Viral RNA from fresh allantoic fluid was extracted by RNA purification kit (TransGen Biotech, Beijing, China) according to the product manual. RT-PCR was conducted with One-Step RT-PCR SuperMix (TransGen Biotech, Beijing, China). Target PCR products were thereafter sequenced by Sangon Biotech Co., Ltd (Shanghai, China) following the purification using DNA Gel Extraction Kit (Axygen Biosciences, Union City, CA, USA).

### Genome Sequence Analysis and Homology Modeling

Nucleotide sequence editing and aa sequence prediction were conducted using BioEdit 7.2.5 and MEGA 7.0 software. To determine the spatial positions of aa mutations, a homology model of NDV HN protein was constructed using the Automated Mode of Swiss-Model software (16). Protein Data Bank (PDB) files with high quality were screened out as previously reported (17). Next, Swiss-Model chose a suitable template (PDB ID: 1e8v.1.A) as the structural basis for homology modeling after automatic filtering. The three-dimensional (3D) structure and positions of hydrogen bond (H-bond) were computed and displayed using the RasMol and PyMol 2.4.0 softwares (18, 19).

### Construction of Genotype III NDV Full-Length CDNAs

**Figure 1:**
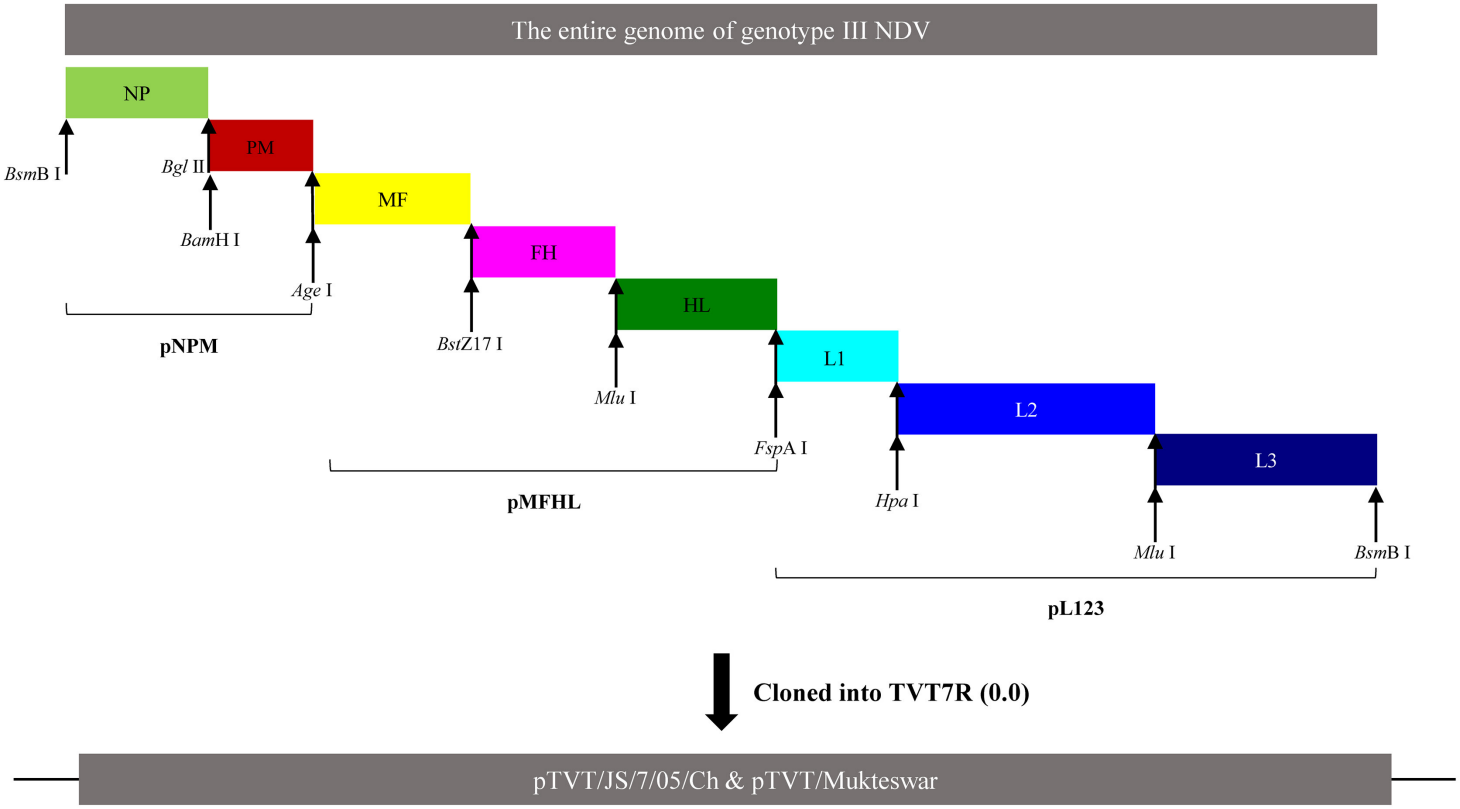
Construction of full-length cDNAs of Mukteswar and JS/7/05/Ch. The NDV genome was divided into eight fragments marked by various colors, including NP, PM, MF, FH, HL, L1, L2, and L3. cDNA fragments were joined at shared restriction sites marked with arrows and then cloned into transcription vectors. The intermediate cDNAs (pNPM, pMFHL, and pL123) and full-length cDNAs of each genotype III NDV were successfully constructed. The full-length cDNAs were thereafter acquired by cloning into the pTVT7R(0.0) vector and named as pTVT/Mukteswar and pTVT/JS/7/05/Ch.

The full-length cDNA clones of genotype III NDVs were amplified using eight pairs of primers (Table 1). As shown in Figure 1, the complete NDV genome was divided into eight fragments including NP, PM, MF, FH, HL, L1, L2, and L3. The eight amplicons were purified and ligated to obtain the full-length tandem genome in the pCR 2.1 vector. The full-length cDNAs were then cloned into the TVT7R (0.0) vector, generating two infectious full-length clones (pTVT/JS/7/05/Ch and pTVT/Mukteswar). These full-length cDNAs were identified by digestion with an *Eco*R I restriction site.

**Table 1:**
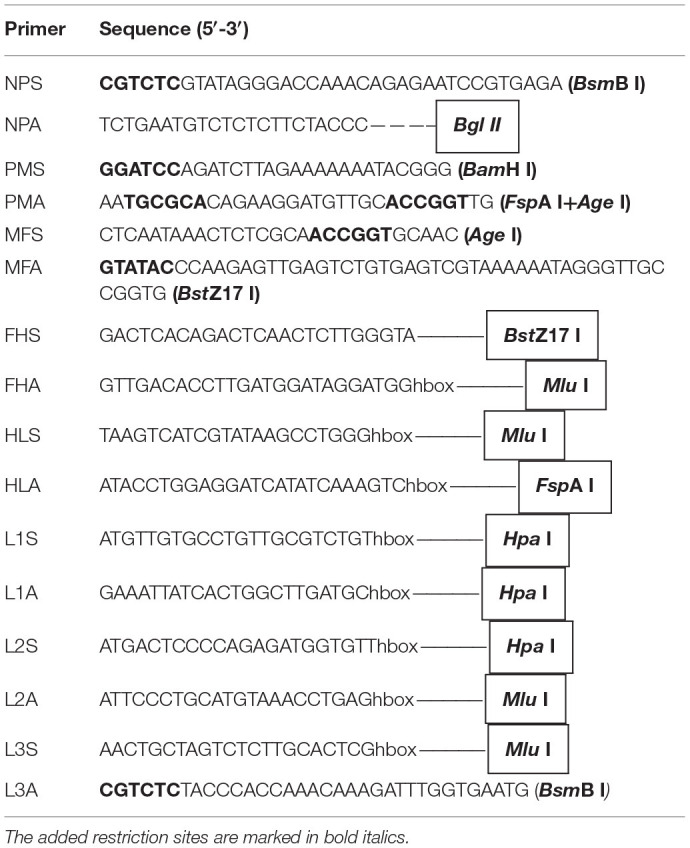
Primer sequences used to construct full-length cDNAs.

*The added restriction sites are marked in bold italics*.

**Figure 2:**
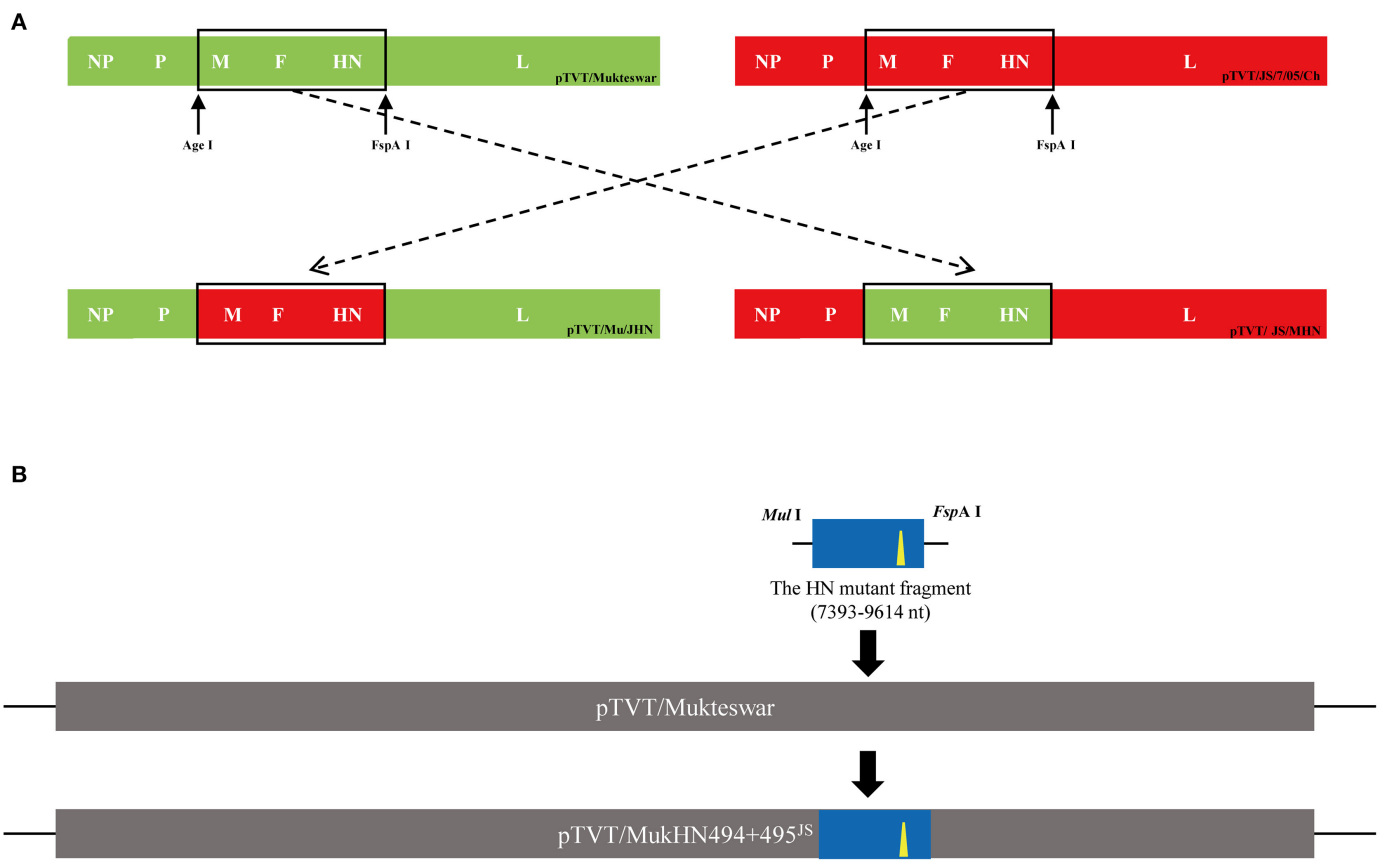
Construction of chimeric NDV cDNAs. **(A)** Schematic representation showed a protocol for the HN exchange between Mukteswar and JS/7/05/Ch. Two restriction sites *Age* I and *Fsp*A I, introduced into the intergenic region, were used to swap the HN gene. The replacement segment included the M, F, HN, and partial L genes. Two HN-replacement chimeric cDNAs were constructed and named as pTVT/Mu/JHN and pTVT/JS/MHN. The locations of the restriction sites were indicated by black arrows. **(B)** Schematic representation of the dual-site mutant cDNA pTVT/MukHN494+495^JS^ construction. The HN mutant fragment was obtained using a fast mutagenesis system, in which dual positions 7,892 and 7,894 of Mukteswar were mutated into those of JS/7/05/Ch. The corresponding DNA segment of pTVT/Mukteswar was then replaced with the dual-site mutant fragment, resulting in the recombinant cDNA pTVT/MukHN494+495^JS^. Restriction sites were shown in the mutant fragment. The nucleotide mutations at positions 7,892 and 7,894 of HN were shown as yellow triangles.

HN gene swapping using the Mukteswar and JS/7/05/Ch genomes as backbones was performed using two restriction enzymes (*Age* I and *Fsp*A I) (Figure 2A). Two chimeric NDV cDNAs (pTVT/JS/MHN and pTVT/Mu/JHN) were further characterized by restriction digestion with *Eco*R I. Additionally, the sequence of Mukteswar was altered by replacement of positions 7,892 and 7,894 with the dual-site mutations of JS/7/05/Ch, yielding the recombinant cDNA pTVT/MukHN494+495^JS^. The cDNA sequence was confirmed by DNA sequencing (Figure 2B).

### Recovery and Characterization of Virus Produced From CDNA

As described previously, the recombinant NDVs were rescued by co-transfecting each full-length cDNA clone with three helper cDNA clones into BSR cells (20). The harvested cell culture supernatants and cell monolayers were injected into the allantoic cavities of SPF embryonated eggs 96 h post-transfection. Allantoic fluid was harvested following embryo death and subjected to hemagglutination and hemagglutination inhibition assays according to standard procedures (21). The rescued viruses were characterized by viral RNA extraction, RT-PCR, and sequencing after passaging in SPF embryonated eggs three times. Finally, the recombinant parental and chimeric viruses were named as rMukteswar, rJS/7/05/Ch, rJS/MHN, rMu/JHN, and rMukHN494+495^JS^, respectively.

The virulence of rescued viruses were determined according to standard procedures, including the intravenous pathogenicity index (IVPI), intracerebral pathogenicity index (ICPI), and mean death time (MDT) tests (21). The 50% embryo infectious doses (EID_50_) of these viruses were determined following the Reed and Muench method.

### Growth Curves

The growth kinetics of recombinant viruses were evaluated in PBMCs. The recombinant viruses were inoculated into cells with an MOI of 0.1 and 1. Cells were thereafter washed three times with PBS to remove unattached viruses and cultured in complete medium at 37°C with 5% CO_2_. The cell supernatant was collected and determined by the 50% tissue culture infective dose (TCID_50_) for indicated time points. The growth curves of NDVs were presented as the line charts drawn by GraphPad Prism 7.00 (Graph Pad Software, Inc., USA).

### Cross Virus-Neutralization (VN) Assay

To investigate whether dual aa mutations A494D and E495K in HN caused antigenic changes of NDV, we carried out the cross-VN test as described by Liu et al. (22). Antisera used in this study were inactivated and provided by our lab. The antisera were prepared from SPF chickens vaccinated with inactivated oil-emulsion rMukteswar, rJS/7/05/Ch and rMukHN494+495^JS^, respectively. These antisera were then serially diluted multiplicatively with PBS and mixed, respectively, with each virus at 100 EID_50_/100μl. The mixtures were incubated at 37°C for 1 h and then injected into SPF embryonated chicken eggs to determine neutralization levels. The 50% endpoints were calculated according to the Reed and Muench method (23). The R value was calculated using the formula of Archetti and Horsfall (24).

### Animal Experiments

To determine the effects of A494D and E495K on pathogenicity *in vivo*, we measured viral loads and inflammatory cytokine expression post-infection. First, groups of 60 1-month-old SPF chickens (20 for each rMukteswar-, rJS/7/05/Ch-, and rMukHN494+495^JS^-infection) were intravenously infected with 10^7^ EID_50_ of virus per chicken. Mock-infected chickens (as negative control) were injected intravenously with an equal amount of phosphate-buffered saline (PBS). Each group was observed twice daily for 10 days. At 12, 24, and 48 h post-infection (hpi), and at 4 and 6 days post-infection (dpi), three chickens from each group were randomly dissected and the spleen, lung, bursa, and thymus were harvested. Duplicate samples were taken from each organ: 0.3 g each for the viral load measurement, and 0.02 g each for the measurement of cytokine levels. These collected organ samples were stored at −70°C until use.

Total RNA of chicken organs was extracted with Trizol reagent (TransGen Biotech, Beijing, China), then synthesizing the cDNA fragments by PrimeScript^TM^ RT Master Mix (Takara, Shiga, Japan) according to product manuals. As for the determination of viral loads, the conserved region of the NDV M gene was amplified using the primers (M-gene F and M-gene R) as shown in Table 2. The amplified M gene was next cloned into the pCMV-vector to serve as the standard plasmid. The copy number was calculated as described previously (25). The cDNA was next subjected to the viral load detection using SYBR Premix reagent (Takara, Shiga, Japan) according to the product manual. The PCR program was set to 30 s at 95°C (pre-denaturation), followed by 40 cycles consisting of 5 s at 95°C and 31 s at 60°C. The ddH_2_O and standard plasmid were used as negative and positive controls for quantitative real-time PCR (qPCR), respectively. As for the detection of cytokine expression, an equal quantity of cDNA (2 μl) of each sample was used for examining the amplification of various cytokines. Real-time PCR was detected by SYBR Color Master Mix for qPCR (Vazyme, Nanjing, China) following the product manual. The expression level of house-keeping gene β-actin was employed for data normalization. Relative gene expression levels were calculated using the 2^−Δ*ΔCT*^ method (26).

**Table 2:** The qPCR primers utilized in this study.

**Gene name**	**Primer sequence (5^**′**^-3^**′**^)**	**Length (bp)**
β-actin F	ATTGTCCACCGCAAATGCTTC	113
β-actin R	AAATAAAGCCATGCCAATCTCGTC	
IL-18 F	AGGTGAAATCTGGCAGTGGAAT	125
IL-18 R	ACCTGGACGCTGAATGCAA	
IL-1β F	GCTCTACATGTCGTGTGTGATGAG	187
IL-1β R	TGTCGATGTCCCGCATGA	
IL-6 F	TTCGCCTTTCAGACCTACCT	213
IL-6 R	TGGTGATTTTCTCTATCCAGTCC	
M-gene F	GCTTGTGAAGGCGAGAGGTG	99
M-gene R	AACCTGGGGAGAGGCATTTG	

### Statistical Analysis

All data are presented as the means ± standard deviations (SD) of three independent replicates from a representative experiment. Statistically significant differences were determined by two-way analysis of variance (ANOVA). All statistics were evaluated using GraphPad Prism 7.00 software (San Diego, CA, USA). *P*-values, <0.05, were considered statistically significant.

## Results

### Analysis of Differential Amino Acid Sites and 3D Protein Structures

**Figure 3:**
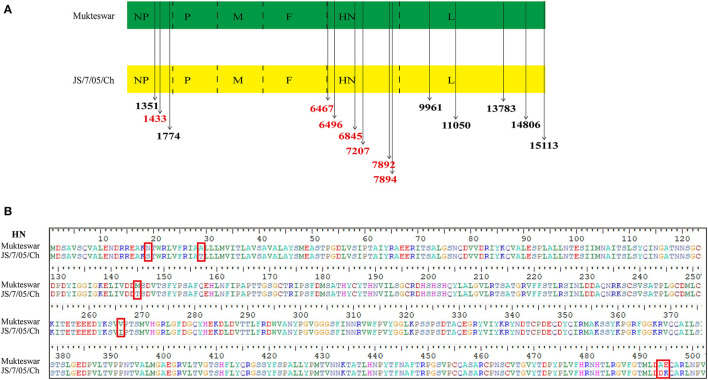
Diagrams showing differential nucleotide and amino acid (aa) sites of the HN protein. **(A)** Two genotype III NDVs showed 14 nucleotide differences in the encoded structural proteins, including NP, HN, and L. The differential nucleotide sites that cause aa variations were marked in red, and others were drawn in black. **(B)** There were six aa mutations in HN proteins between Mukteswar and JS/7/05/Ch, including 19, 29, 145, 266, 494, and 495 aa. The aa mutations in red frames were displayed using BioEdit 7.2.5 software.

**Figure 4:**
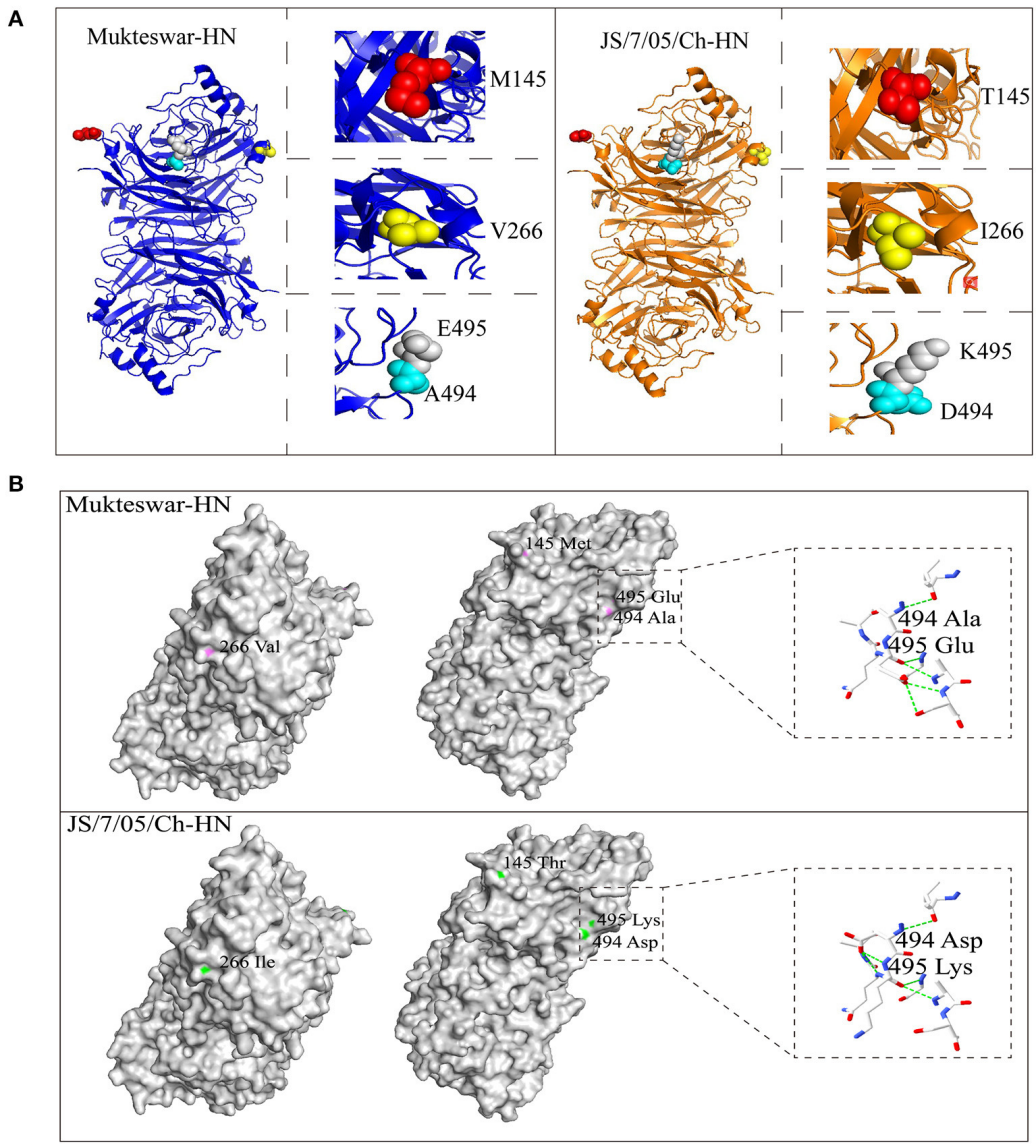
Positions of differential amino acid (aa) residues at the three-dimensional (3D) structure of the HN protein. Homology modeling showed spatial positions of four aa mutations, including 145, 266, 494, and 495 aa. **(A)** The monomer of the HN protein was represented by cartoon mode, including Mukteswar in blue and JS/7/05/Ch in orange. The residues were shown by spheres model in the zoom-in picture, involving 145 aa in red, 266 aa in yellow, 494 aa in cyan, and 495 aa in gray. **(B)** Differential aa residues in the HN protein between Mukteswar and JS/7/05/Ch were marked with violet and green, respectively. Among these aa mutations, A494D and E495K caused H-bond variations drawn by dotted lines in the zoom-in picture. Color coding: blue, nitrogen; red, oxygen; white, carbon. Based on the crystal structure of the HN protein, homology models were carried out using PyMOL 2.4.0 software (PDB ID: 1e8v.1.A).

Fourteen nucleotide differences in the *NP, HN*, and *L* genes were identified (Figure 3A). Seven nucleotide mutations resulted in aa mutations: proline (P) to serine (S) at position 438 (P438S) in NP, and asparagine (N) to S at position 19 (N19S), S to threonine (T) at position 29 (S29T), methionine (M) to T at position 145 (M145T), valine (V) to isoleucine (I) at position 266 (V266I), alanine (A) to aspartic acid (D) at position 494 (A494D), and glutamic acid (E) to lysine (K) at position 495 (E495K) in HN (Figure 3B). Because most aa differences were located in the HN protein, we conducted homology modeling of the 3D structure of HN. The sequence identities between the template (PDB ID: 1e8v.1.A) and the HN protein were higher than 90% and thus suitable for homology modeling. Differential amino acids were marked with various colors in the HN molecular structure as illustrated in Figure 4. Four aa mutations (positions 145, 266, 494, and 495) were shown; positions 19 and 29 were not shown because of the incomplete HN crystal structure. These four aa were differentially distributed at the periphery of the HN dimer. Notably, A494D and E495K changed the positions of H-bonds near these mutations as shown in Figure 4B, which might impact structural stability.

### Generation and Characterization of Recombinant Parental and Chimeric NDVs

**Figure 5:**
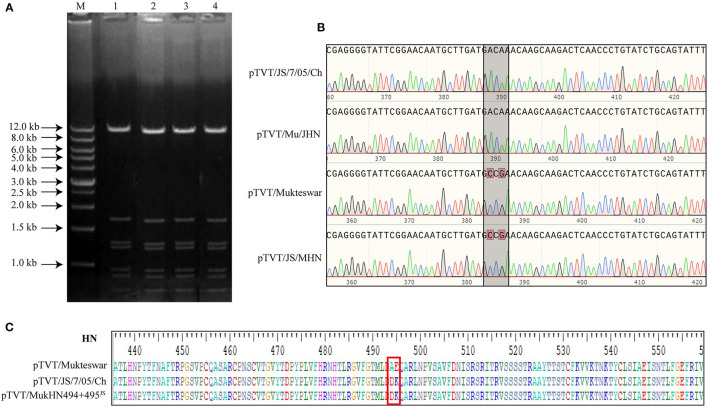
Identification of constructed cDNAs. **(A)** Constructed cDNAs were digested with *Eco*R I. 1: pTVT/JS/7/05/Ch; 2: pTVT/JS/MHN; 3: pTVT/Mukteswar; 4: pTVT/Mu/JHN; M: DNA marker. **(B)** Constructed cDNAs were identified by DNA sequencing. Sequences of RT-PCR products amplified by HLS and HLA were analyzed using BioEdit 7.2.5 software. **(C)** pTVT/MukHN494+495^JS^ showed two aa mutations at positions 494 and 495 compared with pTVT/Mukteswar. Amino acids in NDV HN protein were analyzed by BioEdit 7.2.5 software. The 494 and 495 aa were marked in a red box.

Four full-length NDV cDNAs were successfully constructed and characterized by restriction digestion. As expected, the complete full-length cDNAs were digested into seven bands (0.76, 0.9, 0.95, 1.2, 1.3, 1.7, and 11.0 kb) (Figure 5A). Amplified sequences from pTVT/Mu/JHN and pTVT/JS/MHN using primers of HLS and HLA were identical to the sequences of pTVT/JS/7/05/Ch and pTVT/Mukteswar, respectively (Figure 5B). Four recombinant NDVs (rMukteswar, rJS/7/05/Ch, rMu/JHN, and rJS/MHN) were rescued after the inoculation of cell culture supernatants and cell monolayers into SPF embryonated chicken eggs. Additionally, the recombinant cDNA pTVT/MukHN494+495^JS^ was successfully constructed and confirmed by DNA sequencing (Figure 5C). The dual-site mutant NDV (rMukHN494+495^JS^) was thereafter rescued as described above.

These rescued viruses were subjected to IVPI, ICPI, and MDT assays. As shown in Table 3, genotype III NDVs bearing the mutant HN protein showed significantly elevated IVPI values. The IVPI value for rJS/7/05/Ch was 1.89, significantly higher than that of rMukteswar (0.16). Two HN swapped recombinant viruses (rMu/JHN and rJS/MHN) showed virulence comparable with the parental virus carrying the same HN gene. Briefly, rMu/JHN bearing JS/7/05/Ch-type HN had an IVPI value of 1.77, significantly higher than that of rJS/MHN bearing Mukteswar-type HN (0.12). Moreover, rMukHN494+495^JS^ had a higher IVPI value (1.20) than that of its backbone virus rMukteswar and was more similar in this respect to rJS/7/05/Ch carrying the same mutations at 494 and 495 aa of HN. Furthermore, these recombinant viruses also showed some differential ICPI values. The ICPI values for rJS/7/05/Ch (1.88) and rMu/JHN (1.80) were higher than rMukteswar (1.44) and rJS/MHN (1.46). rMukHN494+495^JS^ also exhibited a higher ICPI value (1.78) than rMukteswar and was closer in this respect to rJS/7/05/Ch. Thus, the mutant HN protein, especially dual aa mutations A494D and E495K in HN, was required for viral virulence, especially after intravenous infection.

**Table 3:** Characterization of the recombinant viruses.

**Virus**	**IVPI score*^**a**^***	**ICPI score*^**b**^***	**MDT (h)*^**c**^***	**EID_**50**_ (0.1ml)*^**d**^***
rMukteswar	0.16	1.44	46.4	10^8.17^
rJS/7/05/Ch	1.89	1.88	46.4	10^7.69^
rMu/JHN	1.77	1.80	48	10^7.72^
rJS/MHN	0.12	1.46	46.4	10^7.83^
rMukHN494+495^JS^	1.20	1.78	46.8	10^7.6^

*^a^Intravenous pathogenicity index (IVPI) tests were determined by intravenously infecting 6-week-old SPF chickens with fresh infective allantoic fluid. The IVPI values of highly virulent strains could reach up to 2.0, while avirulent strains only had IVPI values of 0.0*.

*^b^Intracerebral pathogenicity index (ICPI) tests were determined by intracerebrally infecting 1-day-old SPF chicks with fresh infective allantoic fluid. The pathotype definitions by the ICPI: velogenic strains (1.5–2.0), mesogenic strains (0.7–1.5), and lentogenic strains (0.0 to 0.7)*.

*^c^Mean death time (MDT) tests, the mean time (hours) for the minimum lethal dose to kill all the inoculated embryos, were determined by inoculating fresh allantoic fluid into the allantoic cavities. The pathotype definitions by the MDT: velogenic strains (<60 h), mesogenic strains (60–90 h), and lentogenic strains (>90 h)*.

*^d^egg 50% infective dose*.

### Evaluation of Virus Replication *in vitro*

**Figure 6:**
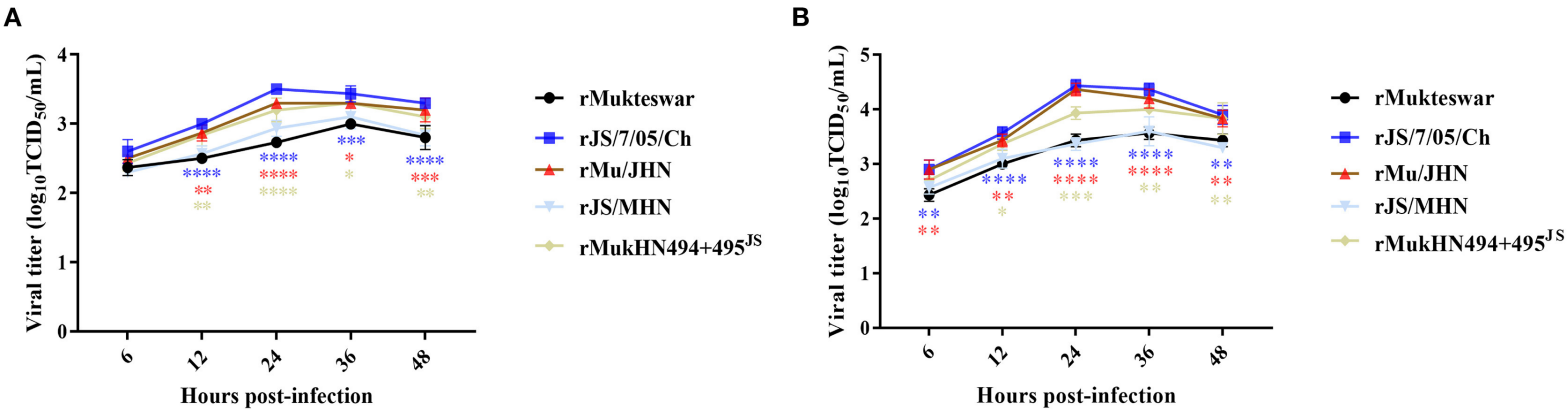
Analyses of growth kinetics of recombinant viruses. The chicken PBMCs were infected with NDVs at an MOI of 0.1 **(A)** and 1 **(B)**. Growth characteristics were evaluated at 6, 12, 24, 36, and 48 hpi. All values of each group were compared with that of rMukteswar. *P < 0.05; **P < 0.01; ***P < 0.001; ****P < 0.0001.

To determine whether the mutant HN protein affected virus replication *in vitro*, we next compared growth curves of the recombinant viruses in chicken cells. Because these genotype III NDVs exhibit significant virulence differences after intravenous inoculation, we further selected chicken PBMCs to determine virus replication. As shown in Figure 6, all of these viruses could grow in PBMCs successfully and showed differential replication abilities. Briefly, rJS/7/05/Ch replicated more efficiently than rMukteswar in PBMCs throughout the infection at multiple MOIs. Similarly, rMu/JHN bearing JS/7/05/Ch-type HN showed the stronger replication ability compared with rJS/MHN bearing Mukteswar-type HN. Notably, the dual-site mutant NDV rMukHN494+495^JS^ exhibited a higher replication level than rMukteswar and was closer to rJS/7/05/Ch in PBMCs. These results demonstrated that dual aa mutations A494D and E495K in HN significantly enhanced NDV replication *in vitro*.

### Evaluation of Effects of A494D and E495K on Antigenic Changes

The cross-VN assay was conducted to evaluate the effects of dual aa mutations A494D and E495K on antigenic diversity. As shown in Table 4, against rNDVs bearing A494D and E495K (rJS/7/05/Ch and rMukHN494+495^JS^), neutralization titers of anti-rMukteswar serum were 1:91 and 1:102, respectively. In addition, the R values between rMukteswar and the two rNDVs bearing A494D and E495K were <0.5 (rMukteswar-rJS/7/05/Ch: 0.26; rMukteswar-rMukHN494+495JS: 0.30). Meanwhile, the R value between rJS/7/05/Ch and rMukHN494+495^JS^ was up to 0.82, indicating no significant antigenic difference between these two viruses. These results suggested that dual aa mutations A494D and E495K in HN induced an obvious significant antigenic difference of NDV.

**Table 4:** Cross virus-neutralization titers for antisera against rMukteswar, rJS/7/05/Ch and rMukHN494+495^JS^.

**Antisera**	**Strains**		
	**rMukteswar**	**rJS/7/05/Ch**	**rMukHN494+495^**JS**^**
Anti-rMukteswar	1:286	1:91	1:102
Anti-rJS/7/05/Ch	1:45	1:205	1:172
Anti- rMukHN494+495^JS^	1:57	1:181	1:228

### Assessment of Viral Loads and Inflammatory Responses in NDV-Infected Chickens

rJS/7/05/Ch induced more severe symptoms and higher mortality compared with rMukteswar. Specifically, rJS/7/05/Ch-infected chickens showed clinical symptoms at 3 dpi, and death occurred at 4 dpi. In contrast, rMukteswar-infected chickens showed only mild lethargy and no deaths were observed. Moreover, rMukHN494+495^JS^-infected chickens exhibited severe clinical signs and mortality similar to those caused by rJS/7/05/Ch-infection.

**Figure 7:**
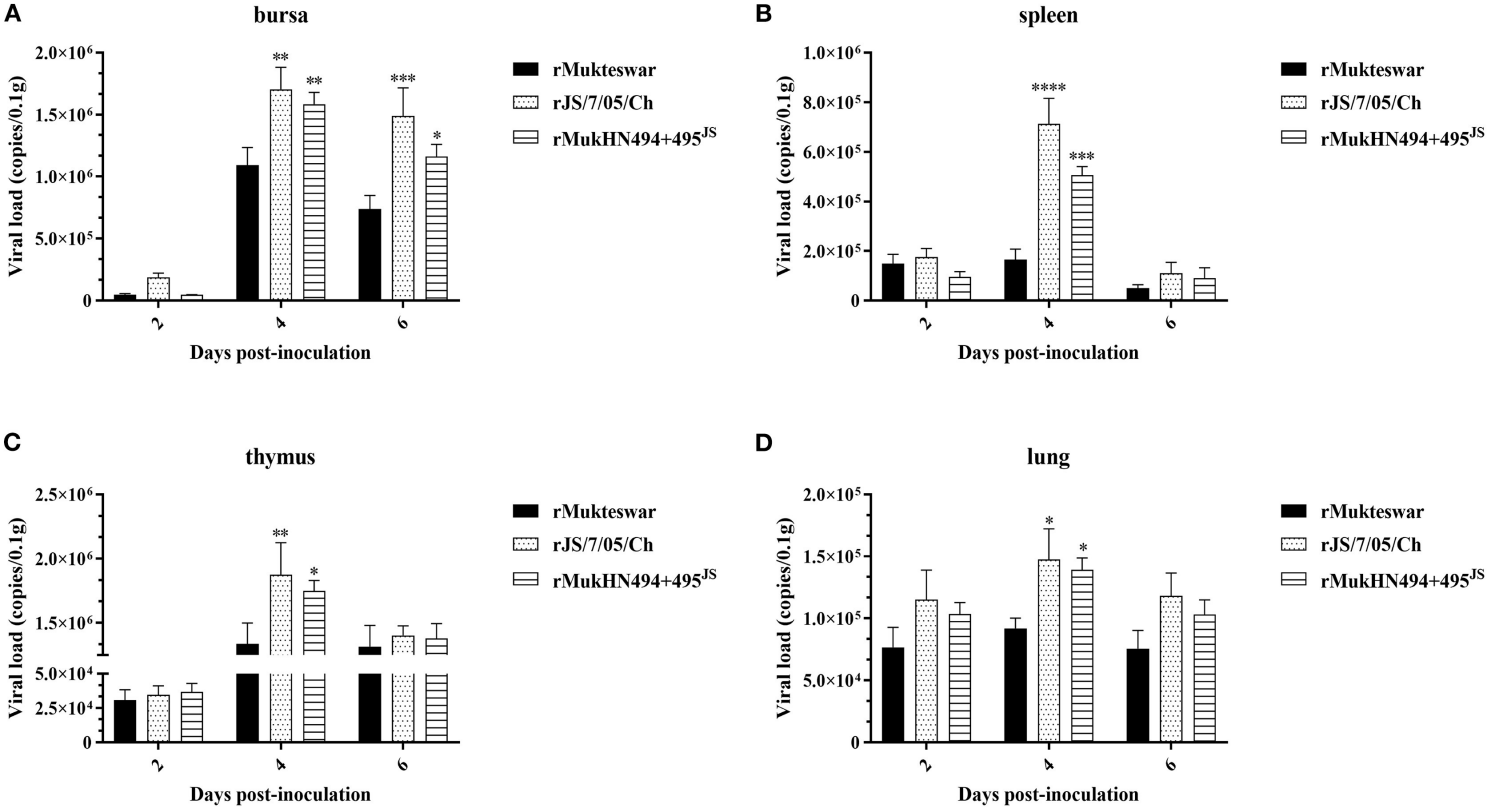
Viral loads in parental and site-mutant rNDV-infected chicken organs. Following infection intravenously with rMukteswar, rJS/7/05/Ch, and rMukHN494+495^JS^, the bursa **(A)**, spleen **(B)**, thymus **(C)**, and lung **(D)** of infected chickens were selected to measure viral loads using real-time PCR. The viral loads in rJS/7/05/Ch- and rMukHN494+495^JS^-infected chickens were compared with that in rMukteswar-infected chickens. *P < 0.05; **P < 0.01; ***P < 0.001; ****P < 0.0001.

To examine whether the A494D and E495K mutations in HN were essential for virus replication *in vivo*, we assessed viral loads in sampled organs of infected chickens (Figure 7). Real-time PCR results showed higher viral loads in rJS/7/05/Ch-infected chickens compared with those in rMukteswar-infected chickens, significantly in the bursa at 4 and 6 dpi and in the spleen, thymus, and lung at 4 dpi. Furthermore, viral loads in all sampled organs were elevated in rMukHN494+495^JS^-infected chickens compared with those in rMukteswar-infected chickens and were similar to those in rJS/7/05/Ch-infected chickens. Besides, rMukHN494+495^JS^ infection exhibited the similar statistical differences to rJS/7/05/Ch infection compared with rMukteswar infection.

**Figure 8:**
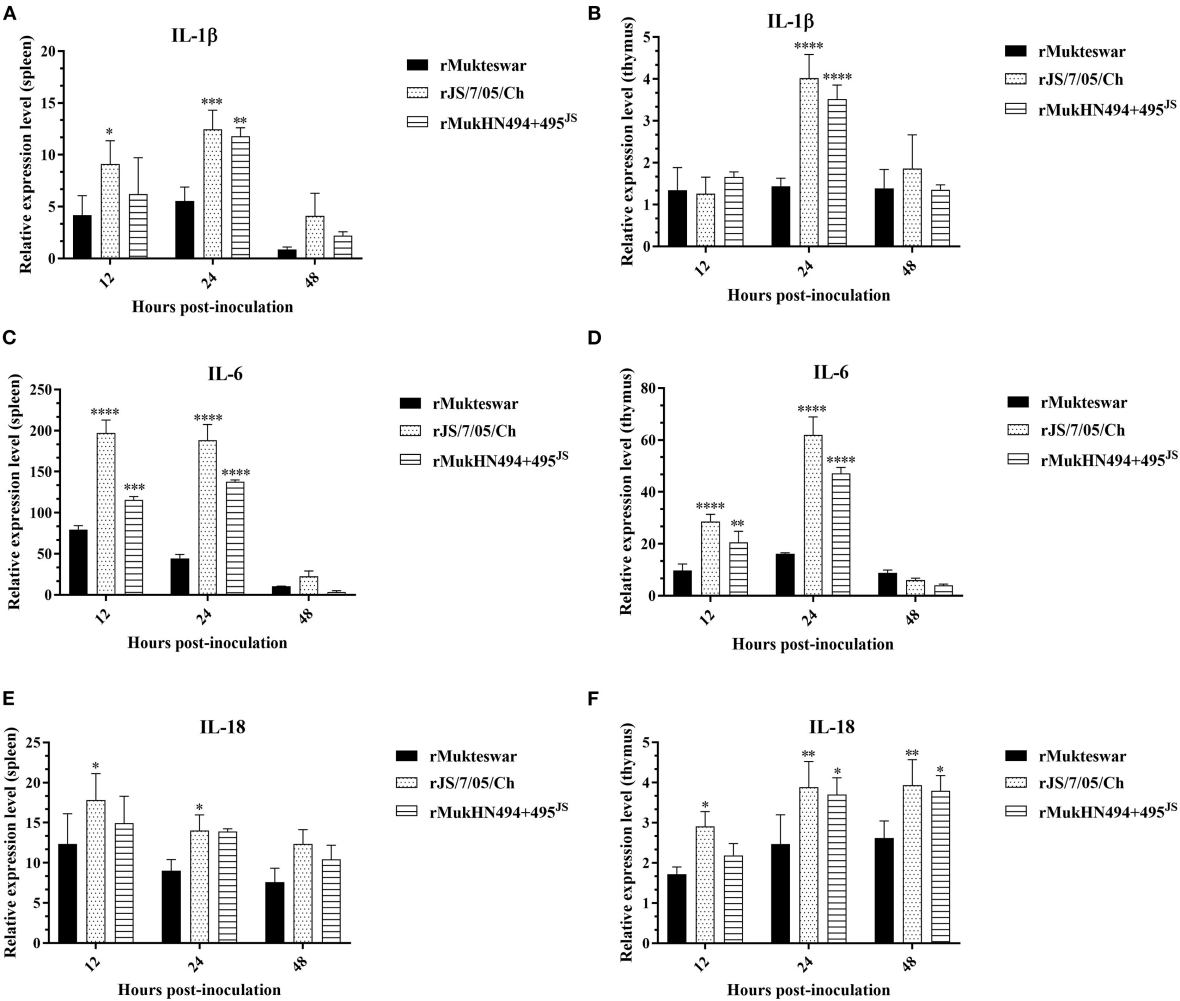
Relative expression of inflammatory cytokine genes in parental and site-mutant rNDV-infected chicken organs. Chickens were infected with rMukteswar, rJS/7/05/Ch, and rMukHN494+495^JS^ through the intravenous route, after which the spleen **(A,C,E)** and thymus **(B,D,F)** were subjected to determine inflammatory cytokine expression using qPCR. The mRNA levels of inflammatory cytokine genes in rJS/7/05/Ch- and rMukHN494+495^JS^-infected chickens were compared with that in rMukteswar-infected chickens. *P < 0.05; **P < 0.01; ***P < 0.001; ****P < 0.0001.

To evaluate the effects of the A494D and E495K mutations on inflammatory responses, we further detected the expression of inflammatory cytokines in rNDV-infected chickens (Figure 8). In the spleen, rJS/7/05/Ch significantly enhanced the mRNA levels of IL-1β, IL-6, and IL-18 in the spleen at 12 and 24 hpi compared with rMukteswar. In the thymus, rJS/7/05/Ch remarkedly strengthened the expression of IL-1β at 24 hpi and IL-6 at 12 and 24 hpi compared with rMukteswar. Additionally, rJS/7/05/Ch induced higher levels of IL-18 than rMukteswar in the thymus throughout the infection. Notably, rMukHN494+495^JS^ induced the similar expression of inflammatory cytokines compared with rJS/7/05/Ch. In brief, rMukHN494+495^JS^ infection significantly upregulated the IL-1β level at 24 hpi and the IL-6 level at 12 and 24 hpi compared with rMukteswar infection in the spleen and thymus. Meanwhile, rMukHN494+495^JS^ also induced higher levels of IL-18 both in the spleen and thymus than rMukteswar throughout the infection, especially at 24 and 48 hpi in the thymus. Overall, these results indicated that dual mutations A494D and E495K in HN enhanced NDV replication and inflammatory responses *in vivo* after intravenous infection.

## Discussion

To control the ND epidemic, vaccination measures are widely applied in China, such as the live vaccine strains La Sota and Mukteswar. The mesogenic vaccine strain Mukteswar was first isolated in India and then attenuated by several passages in chicken embryos (27). Although mesogenic ND vaccines have been abandoned in some countries, Mukteswar is still used for emergency immunization in China because of its strong immunogenicity. RNA viruses are susceptible to mutation and changes in virulence during passaging because RNA polymerases lack proofreading ability. It was previously reported that NDV virulence can be enhanced during serial passage in chickens (28). Moreover, a chicken-derived NDV acquired high pathogenicity during serial passaging in ducks because of the occurrence of aa mutations (29). One virulent genotype III NDV JS/7/05/Ch was isolated from diseased chickens and showed high genomic similarity with Mukteswar (15), suggesting that virulent NDV JS/7/05/Ch may have emerged from Mukteswar during vaccination of poultry. Prior studies of NDV virulence were often performed using lentogenic and mesogenic NDVs, such as La Sota and BC (30). However, ND is commonly caused by virulent NDVs (31), so it is necessary to conduct studies using virulent NDVs as well.

The virulent field strain JS/7/05/Ch shared high identity with the vaccine strain Mukteswar according to whole-genome sequencing. Only 14 nucleotide differences were observed in the NP, HN, and L genes, which caused only seven aa mutations: one in the NP protein and six in the HN protein. HN is made up of four domains: The cytoplasmic domain, the transmembrane region, the stalk region, and the globular head (32, 33). In this study, the aa substitutions in HN between Mukteswar and JS/7/05/Ch were located at positions 19, 29, 145, 266, 494, and 495, which were scattered around various domains of HN. Four aa mutations (positions 145, 266, 494, and 495) were predicted to alter the crystal structure of HN. Structural differences in the HN protein have been reported to affect viral biological activities and virulence (6). Additionally, two aa mutations A494D and E495K in JS/7/05/Ch altered H-bond formation in the HN protein. H-bonds are some of the most important non-covalent interactions in biology and plays roles in stabilizing protein 3D structures and molecular interactions (34). Changes in H-bonding patterns in viral proteins could affect the process of host adaptation (35). Accordingly, we speculate that HN mutations that impact H-bonding may lead to differences in virulence between genotype III NDVs. An increasing number of researches have concentrated on the effects of HN on NDV virulence using reverse genetics technology (6, 10, 36, 37). In this study, two HN-replacement chimeric NDVs (rMu/JHN and rJS/MHN) were generated using a reverse genetics system. rJS/7/05/Ch and rMu/JHN both showed higher IVPI values than rMukteswar and rJS/MHN, indicating that JS/7/05/Ch-type HN enhanced viral virulence during intravenous infection. These findings agree well with previous data suggesting that HN plays a prominent role in NDV virulence following intravenous inoculation (9). NDV HN protein involves in inducing immune protection against virus infection, and thus is more easily to generate antigenic variation under immune pressure (22, 38). Moreover, the 494 and 495 aa of HN have been demonstrated to be located in the antigen epitope and involve in receptor recognition (12, 39, 40). To verify this hypothesis, we conducted the cross-VN test to determine the effects of A494D and E495K on antigenic variation. The results indicated that a significant antigenic difference was observed between rMukteswar and the rNDVs bearing A494D and E495K, while the antigenic difference was not detected between rJS/7/05/Ch and rMukHN494+495^JS^. Therefore, dual mutations A494D and E495K from JS/7/05/Ch-type HN likely cause a significant change in NDV antigenicity. Generally, the antigenic variation probably results in the inefficiency of the vaccine and changes of viral virulence (41, 42). Therefore, we further focused on effects of these two aa mutations on the virulence of the genotype III NDV. As we expected, the dual-site mutant virus bearing A494D and E495K in HN mediated a higher IVPI value. This finding suggests that dual aa mutations A494D and E495K in HN can enhance the virulence phenotype of NDV after intravenous infection. Additionally, PBMCs, distributed in peripheral blood, are widely used as a standard *in vitro* model to study virus infection (43). In this study, A494D and E495K in HN enhanced virus replication in chicken PBMCs, which can be responsible for the increased virulence of NDV after intravenous infection.

To better understand the mechanisms underlying the increased virulence of the genotype III NDV, we selected 1-month-old chickens as models to infect intravenously with recombinant NDVs. Dual aa mutations A494D and E495K in HN significantly boosted NDV replication and pathogenicity *in vivo* following intravenous inoculation. In general, the pathogenicity of virus in animals is thought to be associated with the virus replication level (44). Thus, differential *in vivo* replication abilities of these genotype III NDVs can lead to distinct symptoms and pathogenicity in chickens. Additionally, the host response against NDV infection can influence the pathogenicity of virus (45). In this study, rNDVs bearing D494 and K495 activated inflammatory responses more strongly compared with rMukteswar, which can further explain their severe symptoms and pathogenicity in chickens. Previous researches suggested that efficient viral proliferation can result in severe organ damage and pathogenicity (8), and the strong inflammatory response can contribute to pathogen infection (46, 47). Here, combination of higher viral loads and hyper inflammatory responses may be responsible for the enhanced pathogenicity of the genotype III NDV. Taken together, dual aa mutations A494D and E495K in HN facilitate virus infection and result in higher virulence of the genotype III NDV after intravenous infection. However, the precise mechanism how these aa mutations modulate viral virulence remains to be further investigated.

## Conclusion

Taken together, our study illuminated the molecular mechanisms responsible for increased virulence of the genotype III ND vaccine strain at levels of virus and host. The mutant HN protein of the vaccine strain was identified as the crucial factor in the virulence of NDV after intravenous infection, in which dual aa mutations A494D and E495K in HN played an important role. These findings can be of benefit to the scientific development and application of ND vaccines.

## Data Availability Statement

The datasets presented in this study can be found in online repositories. The names of the repository/repositories and accession number(s) can be found in the article/supplementary material.

## Ethics Statement

The animal study was reviewed and approved by Jiangsu Administrative Committee for Laboratory Animals (Permission number: SYXK-SU-2007-0005).

## Author Contributions

XiuL and SH designed the study. XiaolL and QS performed experiments. XiaolL drafted the manuscript. XiaowL and XW contributed to the revision of the manuscript. All authors read and approved the final manuscript.

## Funding

This work was supported by the National Natural Science Foundation of China: 31873021; by the Earmarked Fund for China Agriculture Research System: CARS-40 and by the Priority Academic Program Development of Jiangsu Higher Education Institutions (PAPD).

## Conflict of Interest

The authors declare that the research was conducted in the absence of any commercial or financial relationships that could be construed as a potential conflict of interest.

## Publisher's Note

All claims expressed in this article are solely those of the authors and do not necessarily represent those of their affiliated organizations, or those of the publisher, the editors and the reviewers. Any product that may be evaluated in this article, or claim that may be made by its manufacturer, is not guaranteed or endorsed by the publisher.
